# Differences among lesions with exon 19, exon 21 EGFR mutations and wild types in surgically resected non-small cell lung cancer

**DOI:** 10.1038/srep31636

**Published:** 2016-08-16

**Authors:** Ying Jin, Ming Chen, Xinmin Yu

**Affiliations:** 1 Department of Medical Oncology, Zhejiang Cancer Hospital, Hangzhou, China; 2 Zhejiang Key Laboratory of Radiation Oncology, Hangzhou, China; 3 Department of Radiotherapy, Zhejiang Cancer Hospital, Hangzhou, China.

## Abstract

The clinical behavior of patients with advanced non-small cell lung cancer (NSCLC) differ between epidermal growth factor receptor (EGFR) exon 19 deletion (Ex19) and EGFR exon 21 L858R mutation (Ex21). This study aimed to evaluate whether these differences exist in surgically resected NSCLC. A total of 198 patients with surgically resected NSCLC harbouring Ex19 (n = 53), Ex21 (n = 51), and EGFR wild-type (Wt) (n = 94) were analyzed. The clinicopathological features, laboratory parameters, recurrent sites and disease-free survival (DFS) were compared according to mutational EGFR status. Ex21 occurred more frequently in female (p < 0.001), never-smokers (p < 0.001), adenocarcinoma (p < 0.001), low grade (p = 0.013) than Wt lesions. Ex19 occurred more frequently in female (p = 0.016), never-smokers (p = 0.008), adenocarcinoma (p < 0.001), low grade (p = 0.025) than Wt lesions. Ex 21 lesions (p = 0.026) had larger lepidic components than Wt lesions. Wt lesions had larger mucinous variant components than Ex21 lesions (p = 0.045) and Ex19 lesions (p = 0.015). Ex21 lesions were associated with lower pretreatment neutrophil: lymphocyte ratio (NLR) than Wt lesions (p = 0.017). The recurrent sites and DFS were similar among patients with Wt, Ex19 and Ex21.

In the last decades, with the progress of targeted therapies in non-small cell lung cancer (NSCLC), the treatment paradigm has been changed for patients with metastatic NSCLC. The epidermal growth factor receptor (EGFR) gene mutations status are with key determinant when using small molecule tyrosine kinase inhibitors (TKIs) for NSCLC patients. EGFR mutations are found in 30% to 50% of lung adenocarcinomas, with the most common mutations being deletion in exon 19 (Ex19 in 45% patients) and a mutation in exon 21 L858R point (Ex21 in 40% patients). Both mutations are referred to as sensitizing EGFR mutations^1^,^2^. Based on the results of eight classic phase 3 randomized trials (IPASS, FIRST-SIGNAL, OPTIMAL, EURTAC, WJTOG3405, NEJ002, LUX-Lung 3, LUX-Lung 6)^3^,^4^,^5^,^6^,^7^,^8^,^9^,^10^ in which both the first-generation (gifitinib, erlotinib) and second-generation (afatinib) of EGFR TKIs have demonstrated increased PFS and response rates than chemotherapy for patients harboring sensitive EGFR mutations, the role of EGFR TKIs have been established as first-line therapy for advanced NSCLC with sensitizing EGFR mutations.

Although the predictive effects of sensitive EGFR mutations-Ex19 and Ex21-are well defined, accumulating data have shown clinical differences between Ex19 and Ex21. Several studies have reported that patients with Ex19 had better survival outcomes than those with Ex21 in advanced NSCLC^11^,^12^. However, little reports has evaluated these differences in surgically resected NSCLC.

In 2011, the International Association for the Study of Lung Cancer (IASLC)/American Thoracic Society (ATS)/European Respiratory Society (ERS) proposed an international classification for lung adenocarcinoma (2011IASLC/ATS/ERS classification)^13^. The system divides adenocarcinoma into adenocarcinoma *in situ* (AIS), minimally invasive adenocarcinoma (MIA), invasive adenocarcinoma (InvAd)-lepidic predominant (LP), InvAd-acinar predominant (AP), InvAd -papillary predominant (PP), InvAd-micropapillary predominant (MP), InvAd-solid predominant (SP), and InvAd-mucinous variant (MV). Previous studies have reported differences features according to the 2011IASLC/ATS/ERS classification between EGFR mutant (Mt) and EGFR wild-type (Wt) resected lung adenocarcinoma. But the conclusions were inconsistent^14^,^15^,^16^.

In this study, we aimed to investigate the differences of clinicopathological features as well as survival outcomes among Ex19, Ex21, and Wt lesions in surgically resected NSCLC. In addition, we analyzed the association between the EGFR mutation status and histological subtypes in the subgroup of lung adenocarcinoma according to the 2011IASLC/ATS/ERS classification.

## Results

### Descriptive characteristics

A total of 198 patients were enrolled in this study: 53 patients (26.8%) with Ex19 lesions, 51 patients (25.8%) with Ex21 lesions, and 94 patients (47.4%) with Wt lesions. The median age at diagnosis was 61 years (range: 26–77). There were 97 (49%) male and 101 (51%) female. The number of patients in stages I–III was 100, 36, and 62 respectively. Thirty-two (16.2%) patients were diagnosed with squamous carcinoma, 161 (81.3%) with adenocarcinoma, and 5 (2.5%) with adenosquamous carcinoma. Thirty-seven (18.7%), 131 (66.2%), and 30 (15.2%) tumors were poorly, moderately, and well differentiated, respectively. One hundred and twelve (56.6%) patients received adjuvant chemotherapy (cisplatinum-based doublets) after operation, and among these patients, the mean cycle of chemotherapy is 3.91 (from 1 to 4). In patients with sensitive mutations, none of them received targeted therapy after operation since there was no indication of EGFR TKIs in the postoperative adjuvant therapy for NSCLC.

### Correlation between EGFR mutation status and clinicopathological features

As shown in Table 1, clinicopathological features were compared among patients with the 3 EGFR statuses. Ex21 occurred more frequently in female (p < 0.001), never-smokers (p < 0.001), adenocarcinoma (p < 0.001), low grade (p = 0.013) than Wt lesions. Ex19 occurred more frequently in female (p = 0.016), never-smokers (p = 0.008), adenocarcinoma (p < 0.001), low grade (p = 0.025) than Wt lesions. Ex21 occurred more frequently in never-smokers (p = 0.041) than Ex19. There were no significant differences in age, T stage, N stage, or tumor size among the 3 groups.

### Correlation between EGFR mutation status and laboratory parameters

As shown in Table 2, laboratory parameters were compared among patients with the 3 EGFR statuses. Ex21 lesions were associated with lower pretreatment NLR than Wt lesions (p = 0.017). While there were no significant differences in CEA, HGB, PLT, or white cell among the 3 groups.

### Correlation between EGFR mutation status and survival outcome

Till December 31, 2015, 74 patients were diagnosed with recurrent or metastatic tumors after surgery. Of the 74 patients, 36 patients had local or lymphatic recurrence, 19 patients had bone metastasis, 14 patients had brain metastasis, 1 patients had adrenocortical metastasis, 2 patients had chest wall metastasis, and 2 patients had liver metastasis. There were no significant differences in recurrent or metastatic sites among the 3 groups (Table 3).

**Figure 1:**
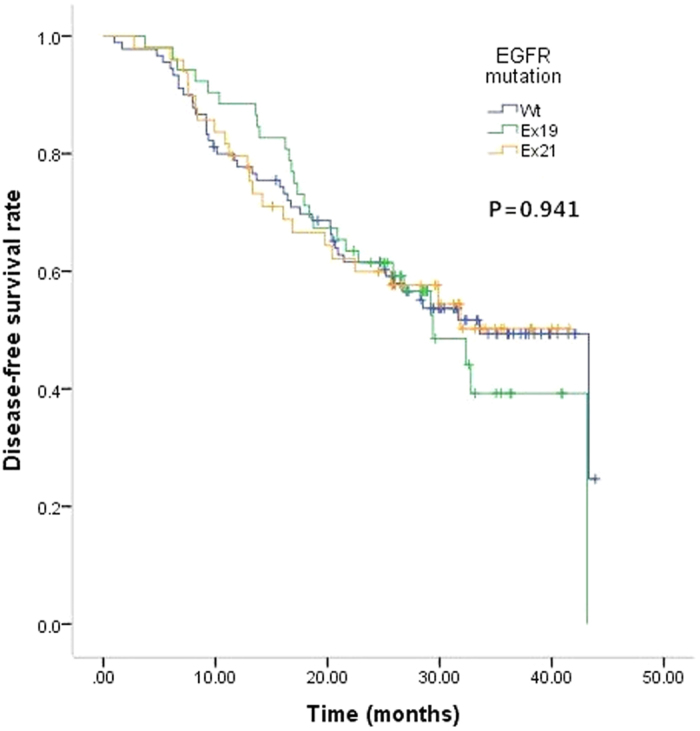
Kaplan-Meier estimates of disease-free survival (DFS) according to EGFR status (Wt, Ex19, and Ex21). The median DFS were 33.6, 29.4, and 25.7 months for patients with Wt, Ex19, and Ex21 lesions, respectively. P = 0.941.

Till December 31, 2015, 191 patients’ DFS data were obtained and 7 patients’ data were censored. The median follow-up was 30.0 months (ranging from 1.7 to 43.9 months). The median DFS were 33.6, 29.4, and 25.7 months for patients with Wt, Ex19, and Ex21 lesions, respectively. There were no significant difference in DFS among the three groups (p = 0.941) (Fig. 1).

### Correlation between EGFR mutation status and pathological subtypes in adenocarcinoma

**Figure 2:**
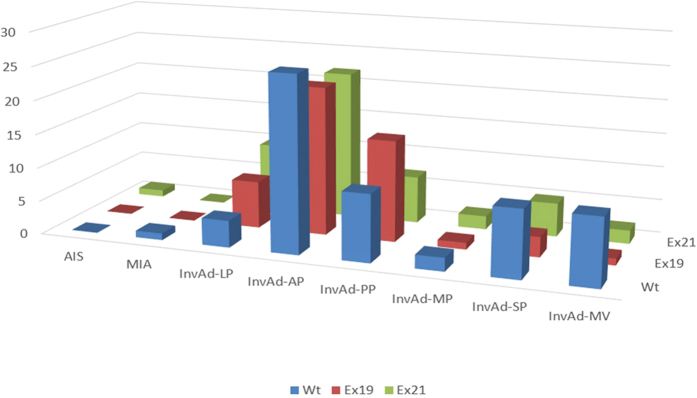
Number of each adenocarcinoma subtype (2011 IASLC/ATS/ERS classification) among the 3 EGFR statuses.

**Figure 3:**
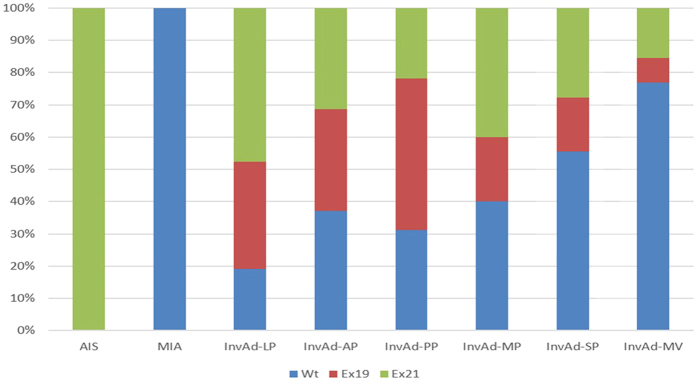
Percentages for the 3 EGFR statuses in each adenocarcinoma subtype based on the 2011 IASLC/ATS/ERS classification.

In the subgroup of 161 patients with adenocarcinoma, we compared the pathological subtypes based on the 2011IASLC/ATS/ERS classification among the three EGFR mutation groups. The most common histological subtype was InvAd-AP (43.5%), followed by InvAd-PP (19.9%), InvAd-LP (13.0%), InvAd-SP (11.2%), and InvAd-MV (7.5%). Other subtypes included one MIA, one AIS and five InvAd-MP. There were 100% of AIS, 81.0% of InvAd-LP, 68.8% of InvAd-PP, 62.9% of InvAd-AP, 60% of InvAd-MP, 44.4% of InvAd-SP, and 23.1% of InvAd-MV subtype were Mt tumors. Ex21 mutations occurred at approximately twice the incidence rate of Ex19 mutations in InvAd-SP and InvAd-MV subtype tumors. Ex19 mutations occurred at approximately twice the incidence rate of Ex21 mutations in InvAd-PP subtype tumors (Figs 2 and 3).

As shown in Table 4, Ex21 lesions (p = 0.026) had larger lepidic components than Wt lesions. Wt lesions had larger mucinous variant components than Ex21 lesions (p = 0.045) and Ex19 lesions (p = 0.015). There were no significant difference in components of other subtypes among the three groups.

## Discussion

At present, we are entering the age of precise medicine for cancer treatment. In recent years, NSCLC, especially lung adenocarcinoma has been found to harbor mutations or rearrangements of specific driver oncogenes, which are used to predict the therapeutic effect of relevant targeted inhibitors^17^,^18^. The most landmark example is that the EGFR mutation status can predict the efficacy of EGFR TKIs. EGFR is a transmembrane receptor tyrosine kinase with extracellular ligand-binding domain, a lipophilic transmembrane region and an intracellular regulatory domain with tyrosine kinase activity. It has been demonstrated that the signaling pathways of EGFR are essential for different cell functions. Mutations of the EGFR genes may result in persistent activation of the tyrosine kinase which could promote proliferation, angiogenesis, invasion, and metastasis of tumor cells in NSCLC^19^,^20^. The two most common sensitive mutations that account for more than 85% of all EGFR gene mutations are Ex19 and Ex21. Several researches have explored clinicopathological differences and prognostic value between Wt and Mt tumors^21^,^22^,^23^,^24^, till now, few studies have compared differences between Ex19 and Ex21. In this study, we for the first time investigate the differences of clinicopathological features as well as survival outcomes among Ex19, Ex21, and Wt lesions in surgically resected NSCLC.

Previous studies have demonstrated that EGFR mutations are commonly observed in a subset of NSCLC patients with the following features: nonsmoker, female, adenocarcinoma, and well- or moderately differentiated tumor cells^25^,^26^. In our study, Ex21 occurred more frequently in female, never-smokers, adenocarcinoma, and low grade tumors than Wt. While the frequencies of female, never-smokers, adenocarcinoma, and low grade tumors with Ex19 tumors were intermediate between the values for Ex21 and Wt tumors. In addition, Ex21 occurred more frequently in never-smokers than Ex19.

Neutrophils in tumor microenvironment have been shown to interact with tumor cells producing cytokines and chemokines, which influence tumor cell growth, angiogenesis and metastasis. In contrast to neutrophils, lymphocytes generally act as the host defense against tumor^27^,^28^. Elevated pretreatment NLR has been proved associated with the poor prognosis of patients with lung cancer^29^. For all we know, so far there has been none of study reported the correlation between NLR and EGFR mutation status. Our study for the first time demonstrated that Ex21 lesions were associated with lower pretreatment NLR than Wt lesions, while the proportion of lower NLR with Ex19 tumors were intermediate between the values for Ex21 and Wt tumors. It is currently believed that inflammatory cells in the tumor microenvironment play a significant role in tumor development. Further evaluation is necessary to examine the interaction and related mechanism between EGFR mutation status and host-derived stromal tissues as well as host immune cells.

The prognostic value of EGFR mutations in resected NSCLC remains controversial. Lee *et al*.^21^ analyzed 117 patients with surgically resected pulmonary adenocarcinoma and found that patients with EGFR mutations had longer DFS than those with Wt. Similarly, D’Angelo *et al*.^30^ analyzed 1118 patients with surgically resected NSCLC and found that patients with EGFR mutations had longer OS than those with Wt. Conversely, some studies revealed that EGFR mutation had no prognostic value for resected NSCLC. Liu *et al*.^23^ investigated 131 patients with resected pulmonary adenocarcinoma and the result showed that there was no significant correlation between EGFR mutation status and DFS, OS. Several article reported different predictive and prognostic value between Ex21 and Ex19 in advanced NSCLC. Liu *et al*.^23^ analyzed 131 patients with resected pulmonary adenocarcinoma and found that patients with Ex19 had longer DFS than those with Ex21. Conversely, Shigemastsu *et al*.^26^ analyzed 62 patients with early-stage NSCLC who underwent resections and found that patients with Ex21 had longer survival time than those with Ex19. In our study, there were no significant difference in DFS among patients with Ex21, Ex19 and Wt. In addition, there were no significant differences in recurrent or metastatic sites among the 3 groups.

Subclassification of lung adenocarcinoma based on the 2011IASLC/ATS/ERS classification had different prognosis. According to previous report, AIS and MIA are classified into low grade; InvAd-LP, InvAd-PP, and InvAd-AP are classified into intermediated grade; and InvAd-SP, InvAd-MP, InvAd-MV are classified into high grade^31^. Yanagawa *et al*.^15^ compared histological subtypes of adenocarcinoma between 131 Mt tumors and 110 Wt tumors. In their report, there were 62% of AIS, 60% of MIA, 77% of InvAd-LP, 49% of InvAd-AP, 50% of InvAd-PP, 28% of InvAd-SP, and 43% of InvAd-MP subtype were Wt tumors, which were incompletely similar to our results. In our study, there were 100% of AIS, 81.0% of InvAd-LP, 68.8% of InvAd-PP, 62.9% of InvAd-AP, 60% of InvAd-MP, 44.4% of InvAd-SP, and 23.1% of InvAd-MV subtype were Mt tumors.

Several studies reported that Mt tumors comprised more commonly the InvAd-LP subtype of lung adenocarcinoma^14^,^16^,^32^. In our study, Mt tumors also more commonly comprised the InvAd-LP subtype than Wt tumors. Several studies reported that Mt tumors comprised more commonly the InvAd-MP subtype of lung adenocarcinoma^33^,^34^. In our study, Wt tumors more commonly comprised the InvAd-MV subtype. Few studies have compared histologic subtypes based on 2011IASLC/ATS/ERS classification between Ex19 and Ex21. Yoshizawa *et al*.^14^ compared 48 Ex19 tumors and 36 Ex21 tumors and found that there were no significant differences in histologic subtypes of adenocarcinoma between Ex19 and Ex21. Villa *et al*.^35^ reported that Ex21 tumors was associated with InvAd-LP subtype when they compared 22 Ex19 tumors and 12 Ex21 tumors. In our study, Ex21 mutations occurred at approximately twice the incidence rate of Ex19 mutations in InvAd-SP and InvAd-MV subtype tumors. Ex19 mutations occurred at approximately twice the incidence rate of Ex21 mutations in InvAd-PP subtype tumors. Both Ex19 and Ex21 lesions had smaller mucinous variant components than Wt lesions. Ex21 lesions had larger lepidic growth components than Wt lesions.

While no significant difference in DFS was observed, the clinicopathological features were different among Wt, Ex19 and Ex21 in early-stage NSCLC who underwent resections. Both Ex19 and Ex21 occurred more frequently in female, never-smokers, adenocarcinoma, low-grade tumors than Wt lesions. Ex21 occurred more frequently in never-smokers than Ex19. Ex21 lesions were associated with lower pretreatment NLR than Wt lesions. Both Ex19 and Ex 21 lesions had smaller mucinous variant components than Wt lesions. Ex 21 lesions had larger lepidic growth components than Wt lesions.

## Methods

### Patients Enrollment

From January 1, 2011 to December 31, 2013, 1775 patients received lung tumors resection with curative intent in Zhejiang Cancer Hospital, Hangzhou, China. Study protocols were approved by the Ethical Review Community of Zhejiang Cancer Hospital. The requirement of informed consent was waived by the committee as it was a retrospective research. All experiments were performed in accordance with the approved guidelines. Subjects eligible for this study had to meet the following criteria: pathologically confirmed NSCLC; surgical specimens for EGFR mutational test were conducted. Patients who received neoadjuvant chemotherapy were excluded. Of the 1775 patients, the EGFR mutation status was analyzed in 209 patients (11.8%). Of the 209 patients, 198 patients were enrolled in this study: 53 patients (26.8%) with Ex19 lesions, 51 patients (25.8%) with Ex21 lesions, and 94 patients (47.4%) with Wt lesions. The 11 patients with other mutation statuses were excluded: 6 patients (2.9%) with G719X mutations in exon 18, 3 patients (1.4%) with L861Q mutations in exon 21, and 2 patients (1.0%) with exon 20 insertions.

### EGFR Mutation Analysis

Genomic DNA was isolated and purified from formalin-fixed paraffin-embedded tissues using the GTpure FFPE Tissue DNA Extraction Kit (GeneTech, Shanghai, China) in accordance with the manufacturer’s instructions. A fragment method was used to detect Ex19/exon 20 insertion. Mt genes were amplified by polymerase chain reaction. To detect exon 18 mutations (G719X), and exon 21 mutations (L858R and L861Q), the Cycleave method was used based on the basic principle of realtime polymerase chain reaction. Each PCR assay contained forward and reverse primers (each 4 pmol), 2 μl template DNA solution, and 2 units of Hot-Start Taq DNA polymerase (Takara, Shiga Japan) in a 40 ml volume. The PCR conditions consisted of initial denaturation at 95 °C for 3 min; 50 cycles of 95 °C for 15 s, annealing at 56 °C for 30 s and 72 °C for 30 s; and final extension at 72 °C for 5 min. The PCR products were sequenced using the Pyrosequencing PyroMark ID system (Qiagen, Hilden, Germany) following the manufacturer’s instructions.

### Clinicopathological Variables and Laboratory Parameters

Clinicopathological data collected for analysis included age at diagnosis, gender, smoking history, tumor size, pathological TNM stage (the seventh edition of the lung cancer staging classification system), pathological types, tumor differentiation, pleural invasion, vessel invasion and histological subtypes of adenocarcinoma according to the 2011IASLC/ATS/ERS multidisciplinary classification of lung adenocarcinoma.

Laboratory data collected for analysis included pretreatment peripheral carcinoembryonic antigen (CEA), haemoglobin (HGB), platlet (PLT), white cell, and neutrophil : lymphocyte ratio (NLR).

### Statistical Analysis

Statistical analysis was performed using SPSS22.0 package. Continuous variables among the 3 EGFR mutation groups were compared using analysis of variance and post hoc comparisons test (Tukey test). We analyzed the association between categorical variables and EGFR mutation status using the Chi-Square test. Whenever it was possible and when the expected value in any of the tests was less than 5, the Fisher exact test was used. A 2-way analysis was performed in all comparisons. Survivals were analyzed using the Kaplan –Meier method and were compared using the log-rank test. Statistical significance was defined as when *P* < 0.05.

## Additional Information

**How to cite this article**: Jin, Y. *et al*. Differences among lesions with exon 19, exon 21 EGFR mutations and wild types in surgically resected non-small cell lung cancer. *Sci. Rep.* **6**, 31636; doi: 10.1038/srep31636 (2016).

## Figures and Tables

**Table 1:** Comparison of clinical backgrounds among the 3 EGFR Statuses in 198 patients with resected non-small cell lung cancer.

Total n = 198	Wt (n = 94)	Ex19 (n = 53)	Ex21 (n = 51)	P values
Age	60.6	59.9	61.9	0.507
Female, %	34 (36.2)	30 (56.6)	37 (72.5)	<0.001
Smoking history, %	57 (60.6)	20 (37.7)	10 (19.6)	<0.001
Tumor size, mm	34.7	33.1	28.6	0.221
T stage, %				0.221
1	31 (33.0)	10 (18.9)	18 (35.2)	
2	54 (57.5)	40 (75.5)	31 (60.8)	
3	7 (7.4)	2 (3.7)	1 (2.0)	
4	2 (2.1)	1 (1.9)	1 (2.0)	
N stage, %				0.147
0	50 (53.2)	24 (45.3)	33 (64.7)	
1	22 (23.4)	8 (15.1)	4 (7.8)	
2	22 (23.4)	21 (39.6)	14 (27.5)	
Pathological types, %				<0.001
Squamous	30 (31.9)	1 (1.9)	1 (2.0)	
Adenocarcinoma	63 (67.0)	49 (92.5)	49 (96.0)	
Adenosquamous	1 (1.1)	3 (5.6)	1 (2.0)	
Grade, %				0.005
High	22 (23.4)	7 (13.2)	8 (15.7)	
Intermediate	65 (69.2)	36 (67.9)	30 (58.8)	
Low	7 (7.4)	10 (18.9)	13 (25.5)	
Pleural invasion, %	44 (46.8)	32 (60.4)	33 (64.7)	0.082
Vessel invasion, %	20 (21.3)	9 (17)	4 (7.8)	0.121

**Table 2:** Comparison of laboratory backgrounds among the 3 EGFR Statuses.

	Wt	Ex19	Ex21	P values
CEA (ng/ml)				0.698
>5	26 (29.2)	17 (33.3)	15 (31.9)	
≤5	63 (70.8)	34 (66.7)	32 (68.1)	
HGB (g/dl)				0.419
≥12	79 (84.0)	44 (83.0)	40 (78.4)	
<12	15 (16.0)	9 (17.0)	11 (21.6)	
PLT (109/l)				0.149
≥300	9 (9.6)	2 (3.8)	2 (3.9)	
<300	85 (90.4)	51 (96.2)	49 (96.1)	
White cell (109/l)				0.162
<4	5 (5.3)	2 (3.8)	7 (13.7)	
4~10	87 (92.6)	50 (94.3)	43 (84.3)	
>10	2 (2.1)	1 (1.9)	1 (2.0)	
NLR				0.02
≤2.2	50 (53.2)	34 (64.2)	37 (72.5)	
>2.2	44 (46.8)	19 (35.8)	14 (27.5)	

**Table 3:** Comparison of recurrent or metastatic sites among the 3 EGFR Statuses.

	Wt	Ex19	Ex21	P value
Local or lymphatic recurrence				0.601
present	18 (51.4%)	12 (52.2%)	6 (37.5%)	
absent	17 (48.6%)	11 (47.8%)	10 (62.5%)	
Bone metastasis				0.529
present	9 (25.7%)	4 (17.4%)	6 (37.5%)	
absent	26 (74.3%)	19 (82.6%)	10 (62.5%)	
Brain metastasis				0.333
present	5 (14.3%)	5 (21.7%)	4 (25.0%)	
absent	30 (85.7%)	18 (78.3%)	12 (75.0%)	
Adrenocortical metastasis				0.745
present	0 (0%)	1 (4.3%)	0 (0%)	
absent	35 (100%)	22 (95.7%)	16 (100%)	
Chest wall metastasis				0.180
present	2 (5.7%)	0 (0%)	0 (0%)	
absent	33 (94.3%)	23 (100%)	16 (100%)	
Liver metastasis				0.661
present	1 (2.9%)	1 (4.3%)	0 (0%)	
absent	34 (97.1%)	22 (95.7%)	16 (100%)	

**Table 4:** The association between adenocarcinoma histological subtypes and mutational statuses of EGFR in 161 patients with adenocarcinoma.

Total n = 161	Wt (n = 63)	Ex19 (n = 49)	Ex21 (n = 49)	P values
AIS				0.190
0	63 (100)	49 (100)	48 (98.0)	
1	0 (0)	0 (0)	1 (2.0)	
MIA				0.271
0	62 (98.4)	49 (100)	49 (100)	
1	1 (1.6)	0 (0)	0 (0)	
InvAd-LP				0.028
0	59 (93.7)	42 (85.7)	39 (79.6)	
1	4 (6.3)	7 (14.3)	10 (20.4)	
InvAd-AP				0.902
0	37 (58.7)	27 (55.1)	27 (55.1)	
1	26 (41.3)	22 (44.9)	22 (44.9)	
InvAd-PP				0.076
0	53 (84.1)	34 (69.4)	42 (85.7)	
1	10 (15.9)	15 (30.6)	7 (14.3)	
InvAd-MP				0.812
0	61 (96.8)	48 (98.0)	47 (96.9)	
1	2 (3.2)	1 (2.0)	2 (3.1)	
InvAd-SP				0.258
0	53 (84.1)	46 (93.9)	44 (89.8)	
1	10 (15.9)	3 (6.1)	5 (10.2)	
InvAd-MV				0.017
0	53 (84.1)	48 (98.0)	47 (95.9)	
1	10 (15.9)	1 (2.0)	2 (4.1)	
